# Complex *Multi*-*Block Analysis* Identifies New Immunologic and Genetic Disease Progression Patterns Associated with the Residual β-Cell Function 1 Year after Diagnosis of Type 1 Diabetes

**DOI:** 10.1371/journal.pone.0064632

**Published:** 2013-06-05

**Authors:** Marie Louise Max Andersen, Morten Arendt Rasmussen, Sven Pörksen, Jannet Svensson, Jennifer Vikre-Jørgensen, Jane Thomsen, Niels Thomas Hertel, Jesper Johannesen, Flemming Pociot, Jacob Sten Petersen, Lars Hansen, Henrik Bindesbøl Mortensen, Lotte Brøndum Nielsen

**Affiliations:** 1 Department of Pediatrics, Herlev Hospital, Faculty of Health Science, University of Copenhagen, Copenhagen, Denmark; 2 Department of Food Science, Quality and Technology, Copenhagen, Denmark; 3 Department of Pediatrics, Skejby University Hospital, Aarhus, Denmark; 4 Department of Pediatrics, Kolding Hospital, Kolding, Denmark; 5 H.C. Andersen Childrens Hospital, Odense University Hospital, Odense, Denmark; 6 Department of Biochemistry, Diagnostic Unit, Glostrup University Hospital, Glostrup, Denmark; 7 Diabetes Pharmacology and Bioanalysis, Novo Nordisk A/S, Måløv, Denmark; University of Siena, Italy

## Abstract

The purpose of the present study is to explore the progression of type 1 diabetes (T1D) in Danish children 12 months after diagnosis using *Latent Factor Modelling.* We include three data blocks of dynamic paraclinical biomarkers, baseline clinical characteristics and genetic profiles of diabetes related SNPs in the analyses. This method identified a model explaining 21.6% of the total variation in the data set. The model consists of two components: (1) A pattern of declining residual β-cell function positively associated with young age, presence of diabetic ketoacidosis and long duration of disease symptoms (*P* = 0.0004), and with risk alleles of *WFS1*, *CDKN2A/2B* and *RNLS* (*P* = 0.006). (2) A second pattern of high ZnT8 autoantibody levels and low postprandial glucagon levels associated with risk alleles of *IFIH1*, *TCF2*, *TAF5L*, *IL2RA* and *PTPN2* and protective alleles of *ERBB3* gene (*P* = 0.0005). These results demonstrate that *Latent Factor Modelling* can identify associating patterns in clinical prospective data – future functional studies will be needed to clarify the relevance of these patterns.

## Introduction

T1D is the end result of T-cell mediated autoimmune destruction of the pancreatic β-cells. Several studies have described the natural history of T1D with respect to β-cell failure and glycaemic control in order to understand disease progression [1]–[3]. In those studies the predominant factors associated with a rapid loss of residual β-cell function are young age [1], [2] and severe diabetic ketoacidosis (DKA) at diagnosis [2], [4]. The causal effect of autoantibodies on residual β-cell function remain unclear as conflicting results are reported [2], [5]. However, a positive association between the arginine variant of the ZnT8 autoantibodies (ZnT8Arg) and the residual β-cell function has recently been reported [6]–[8]. Genome wide association studies (GWAS) have identified in excess of 40 regions with significant association to T1D, but the functionality of these genes in disease mechanisms is not addressed by GWAS studies. Few of the T1D susceptibility genes (*INS* and *PTPN22* genes) have so far been associated with residual β-cell function and glycaemic control during the first year after diagnosis in newly diagnosed children with T1D [9], [10]. Thus, although the residual β-cell function has been extensively studied, individual variation remains to be explained.

The complexity of T1D pathogenesis advocates for new modelling methods in biomedical systems of equivalent complexity [11], [12], especially regarding gene-gene interactions (epistasis) [13]. The usage of *Latent Factor Modelling* for analysis of complex data is an emerging field originating from genomics, metabolomics and chemometric sciences and is gaining acceptance in clinical research [14], [15]. By applying the *multi-block* approach when analysing closely monitored clinical cohorts instead of classical regression analyses we may identify new associations between biomarker patterns related to disease progression, corresponding baseline characteristics and gene-gene interactions [16].

The aim of this study was to investigate patterns of clinical-, paraclinical- and genetic characteristics during the first 12 months after diagnosis in a Danish cohort of 129 children with newly diagnosed T1D by applying *Latent Factor Modelli*ng.

## Materials and Methods

### Subjects

The Danish Remission Phase Study is a prospective long-term observational study conducted in four paediatric departments. A total of 129 children and adolescents aged less than 17 years with newly diagnosed T1D were enrolled in the study from April 2004 to August 2006 and followed for 12 months from onset of T1D, defined as the first insulin injection. The detailed study design has previously been described by Andersen et al [8]. The study was performed according to the criteria of the Helsinki II Declaration and was approved by the Danish National Committee on Biomedical Research Ethics (Journal number: H-KA-04010-m). Older patients and all parents or guardians gave written informed concept. Data were transmitted anonymously by the centers; patients were identified by center number and patient code.

### HbA_1c_, Insulin Dose Adjusted HbA_1c_ (IDAA1c) and Partial Remission

**HbA_1c_** was measured at disease onset, 1, 3, 6, 9, and 12 months after diagnosis (±1 week). Capillary samples for HbA_1c_ analysis were centrally measured using Bio-Rad HbA_1c_ sample preparation kit (Bio-Rad Laboratories, Munich, Germany) as described by DCCT [1]. Normal range for the assay was 4.4–6.3% (about 0.3% higher than the DCCT method). Daily insulin dose (U/kg) was recorded 1, 3, 6, 9 and 12 months after diagnosis. **IDAA1c** was calculated as IDAA1C = HbA_1c_ (percent)+[4 x insulin dose (units per kilogram per 24 h)]. **Partial remission** was defined as an IDAA1C ≤9 as described by Mortensen et al [17].

### Residual β-cell Function

After 1, 3, 6 and 12 months of diabetes (±1week) a mixed meal tolerance test (MMTT) was performed to stimulate endogenous C-peptide and proinsulin release as previously described [8]. The included children received 6 ml/kg (maximum 360 ml.), according to DCCT standards [1], of BOOST® Original Drink (237 ml or 8 FL OZ containing 41 g carbohydrate, 10 g protein and 4 g fat, 240 kcal in all from Novartis Medical Health, Inc., Minneapolis, MN, USA (www.boost.com/nutritional-drinks/boost-original). Serum samples were stored on dry ice at −20 °C until shipment to Steno Diabetes Centre. **Stimulated serum C-peptide** was analysed by a fluoroimmunometric assay as previously described [2]. **Stimulated serum proinsulin** was analysed by a sandwich ELISA assay as described by Kaas et al [18]. There were 129 patients who contributed with at least one measurement.

### Glucagon, Glucose-dependent Insulinotrophic Polypeptide (GIP) and Glucagon-like Peptide-1 (GLP-1)

**Glucagon, GIP and GLP-1** were all measured centrally after extraction of plasma with 70% ethanol (vol/vol, final concentration) [19]. There were 129 patients who contributed with at least one measurement.

### Autoantibodies

The conventional T1D autoantibodies were measured in serum samples: **Islet Cell autoantibodies** (ICAs) by use of immunofluorescence assay [20]; **insulin autoantibodies** (IAAs), antibodies against **Glutamic Acid Decarboxylase** (GADAs) and **Insulinoma-associated Antigen-2** (IA-2A) by use of specific radiobinding assays [21]. The cut-off values for ICA, IAA, GADA and IA-2A positivity were 2.5 Juvenile Diabetes Foundation units (JDFU), 2.80 relative units (RU), 5.36 RU and 0.43 RU, respectively. **Zinc transporter 8 autoantibodies (ZnT8Abs)** were measured on serum samples by use of specific radio binding assays (RBA) for each variant (arginine (ZnT8Arg), tryptophan (ZnT8Trp) and glutamine (ZnT8Gln)) and the triple mix assay (ZnT8tripleAb) as described previously [8]. ZnT8Ab positivity was defined as titer values ≥ 60 U/ml, 58 U/ml and 65 U/ml and 58 U/ml for ZnT8Arg, ZnT8Trp, ZnT8Gln and ZnT8tripleAb, respectively. There were 129 patients who contributed with at least one measurement.

### HLA Typing

Time-resolved fluorometry was used for identification of HLA-DQB1 alleles (02, 0301, 0302, 0304, 0602, 0603, and 0604) as described in details [22]. The HLA-DQB1 genotyping risk score was as follows: 'very high risk' included only DQB1*0302-DQB1*02 heterozygous individuals, 'high risk' included DQB1*0302/*0302, 0302/X, 02/02, and 02/X, 'moderate risk' included DQB1*0302/0301, 0302/0603, 02/0301, 02/0603, and XX, and 'low risk' included DQB1*0302/0602, 02/0602, 0301/X, 0603/X, where X are all other DQB1 alleles. Patients were subdivided into three HLA risk groups (very high, high and moderate/low) according to their HLA-DQB1 genotype [23]. 125 children were HLA genotyped, the genotype distribution was: very high risk n = 48, high risk n = 55 and moderate/low risk n = 22.

### Genotyping

In total 125 children were genotyped for 51 single nucleotide polymorphisms (SNPs) from different genomic loci chosen from T1D and T2D GWAS. Genotyping was done using Taqman allele discrimination (KBioscience, Hoddesdon, UK). The 31 selected T1D SNPs were: [24]: *INS* (rs3842753 and rs689), *PTPN22* (rs2476601), *PTPN2* (rs478582 and rs1893217), *IFIH1* (rs1990760), *IL2RA* (rs11594656), *KIAA0350* (rs12708716), *SHSB3* (rs3184504), *ERBB3* (rs2292239), *TAF5L* (rs3753886), *ICAM1* (rs1799969), *SORCS1* (rs1358030), *UBASH3A* (rs9976767), *BACH2* (rs3757247), *CTSH* (rs3825932), *C1QTNF6* (rs229541), *IL6* (rs1800795), *PDCD1* (rs11568821), *BNC2* (rs566369), *IL10* (rs3024505), *IL7* (rs6897932), *TNFAIP3* (rs2327832), *SKAP2* (rs7804356), *CTRB1/CTRB2* (rs7202877), *GSDMB/ORMDL3* (rs2290400), *CTLA4* (rs231775 and rs3087243), *RNLS* (rs10509540), *GLIS3* (rs7020673), *PRKCQ* (rs11258747). The 20 selected T2D SNPs were: [25]: *SLC30A8* (rs13266634), *KCNJ11* (rs5215), *TCF7L2* (rs7901695 and rs7903146), *CDKN2A/2B* (rs564398 and rs10811661), *IGFBP2* (rs4402960), *CDKAL1* (rs10946398), *HHEX/IDE* (rs5015480 and rs1111875), *WFS1* (rs10010131), *ADAMTS9* (rs4607103), *PPARG* (rs1801282), *THADA* (rs7578597), *CDC123/CAMK1D* (rs12779790), *FTO* (rs9939609), *JAZF1* (rs864745), *NOTCH2* (rs10923931), *TSPAN8/LGR5* (rs7961581) and *HNF1B* (rs4430796).

### Statistical Methods

#### Conventional statistical methods

Data are descriptively presented as median and range for non-normally distributed parameters and mean ± standard deviation (SD) for normally distributed parameters. Non-normally distributed parameters were analysed on logarithmic scale. The analyses were performed using SAS (version 9.2, SAS Institute; Cary, NC, USA) and R (http://mirrors.dotsrc.org/cran/).

#### Latent factor models for analysis of complex data – multi-block approach

**Figure 1:**
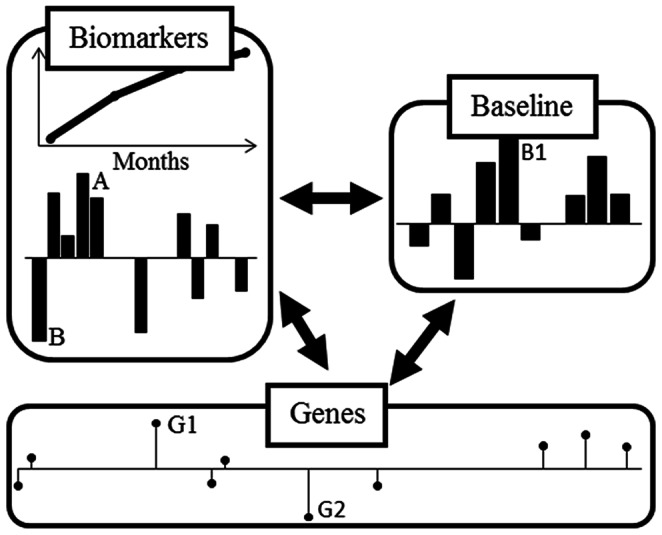
Diagram of a single factor/component from a -block model examining biomarkers over time (Biomarkers) in relation to baseline characteristics (Baseline) and Genetic background (Genes). The pattern indicates that e.g. the biomarker *A* increases- and the biomarker *B* decreases over time. This pattern is e.g. related to high values of the baseline characteristics *B1* and high number of risk alleles for gene *G1* and low number of risk alleles for gene *G2.*

The data are organized as three individual data blocks schematized generically in Figure 1: Block **I**: Paraclinical markers such as number of insulin injections, fasting blood glucose, stimulated blood glucose (SBG), daily insulin dose per kg, body mass index (BMI), HbA_1c_, IDAA1c, insulin antibodies, autoantibodies: GADA, ICA, IA-2A, ZnT8Arg, ZnT8Trp, ZnT8Gln and ZnT8tripleAB and serum level of stimulated: C-peptide, proinsulin, glucagon, GIP and GLP-1 measured 1, 3, 6 and 12 months after diagnosis. Block **II**: Clinical and paraclinical markers registered at onset (baseline): Number of weeks before diagnosis with polyuria and polydipsia, pubertal status, blood glucose, standard bicarbonate (HCO_3_^-^), gender, age, DKA (HCO_3_^-^ ≤15 mmol/L), severe DKA (HCO_3_^-^ ≤5 mmol/L), HLA risk groups and HbA_1c_. Block **III**: T1D and T2D related genetic polymorphisms as described above.

The aim of the analysis was to extract biologically intuitive patterns reflected in the different data blocks. We used a multi-block procedure, *Coupled Matrix Tensor Factorization* [26], [27], to derive common components/factors. The method is an extension of the well-known methods for factorization of matrices (*Latent Factor Models*), *Principal Component Analysis* (PCA), and higher order arrays, *PARAallel FACtor analysis* (PARAFAC). The large number of variables in population-based cohorts is significantly prone to spurious discoveries, for which reason correction for multiple testing is often applied, e.g. Bonferroni correction. The *Latent Factor Modelling* account for multiple testing by reducing the dimensionality in a way similar to principal component analysis [28].

In order to simplify the factors a sparsity constraint was imposed with the result that variables with small contribution are set to zero. Hereby the individual factors only reflect a subset of the variables and are therefore easier to interpret. Thus, from the chosen model based on the three data blocks a number of relevant components are extracted. Each component describes a certain pattern of variation and may be reflected in several modes (biomarkers over time, baseline characteristics and genes). Therefore, all components are parts of the *same* model reflecting different association patterns between the data blocks. Examination of the biomarker mode reveals *which* biomarkers are related to the component, and examination of the time mode reveals *how* this pattern progress over time, see Figure 1 ‘Biomarkers’. Here variable A has a high positive so called loading and B a high negative loading; this means that these two variables, in this pattern, are opposite correlated. The inclusion of the timely development indicates that A increases and B decreases over time. Further, the pattern is extracted such that profiles in ‘Baseline characteristics’ and ‘Genes’ are simultaneously estimated. In figure 1 ‘Genes’, G1 is therefore positively associated- whereas G2 is negatively associated with this pattern in the biomarker development. Likewise baseline variable B1 is positively associated with the pattern (to highlight a few).

#### Validation

The multi-block model was *internal validated* by pattern to pattern association via random permutation testing, which estimates the significance of the associations between the data-blocks in the true model.

The multi-block model was validated further by the use of two different approaches for *external validation*: i) split half consistency and ii) replication in an independent cohort. A split half analysis mitigates the process of estimating the same model in two independent datasets by splitting the data into two equally sized portions followed by building of two independent models. These are compared in terms of pattern estimates by either correlation coefficients (for baseline and biomarker variables) or selectivity patterns for genes (by homogeneity testing). Split half analysis is based on models built on reduced data with a resulting loss of statistical power. Optimally the results can be reproduced in an independent cohort. The Hvidoere Remission Phase Cohort including 275 newly diagnosed children with T1D collected through 18 paediatric centres in Europe and Japan represent an independent, but slightly different cohort [2]. The same blood samples were collected as for the cohort presented in this study (the Danish Remission Phase Cohort) 1, 6 and 12 months after diabetes onset. An independent model is built on these data and compared to the model built on the Danish Remission Phase Cohort in a similar fashion as for the split half analysis.

For further methodological details see appendix S1.

## Results

### Demographic and Clinical Characteristics

Data were obtained from 129 patients, 63 girls (mean age 10.2 yrs) and 66 boys (mean age 9.9 yrs), 95% were Caucasian. Clinical information including anthropometric data, ethnicity, pubertal status, symptoms prior to diagnosis and metabolic status (DKA, stimulated blood glucose and HbA_1c_) are summarized in Table 1 according to age group.

**Table 1:** Clinical and demographic data at onset and 1 month (^‡^) after diagnosis by age groups: *P<0.05.

	Age <5 yrs	5≥ Age <10 yrs	Age ≥10 yrs
Number (male/female)	14/5*	14/22	38/36
Mean age (range) (yrs)	3.2 (0.6–4.9)	7.9 (5.2–9.8)	12.8 (10.1–16.6)
Mean BMI (range) (kg/m^2^)^‡^	16.3 (13.4–20.4)	16.6 (13.4–20.6)	19.5 (14.6–29.9)
Prepubertal (%)	19 (100)	34 (97.1)	21 (36.8)
White Caucasian (%)	19 (100)	33 (91.7)	71 (97.3)
Family history of T1D (%)	5 (26.3)	2 (5.6)	9 (12.2)
Mean duration of polyuria (weeks)	3.7 (1–14)	3.4 (1–16)	4 (0–24)
Mean duration of polydipsia (weeks)	3.7 (1–14)	3.5 (1–16)	3.9 (0–24)
DKA at diagnosis (%)	5 (26)	7 (20)	7 (10)
Mean HbA_1c_ (SD, range) (%)	9.73 (1.16, 7–11.6)	11.35 (1.86, 7.5–14.5)	12.24 (2.31, 7.8–18.5)
Mean blood glucose (SD) (mmol/liter)	31.8 (10.1)	27.1 (10.1)	24.9 (6.8)

**Figure 2:**
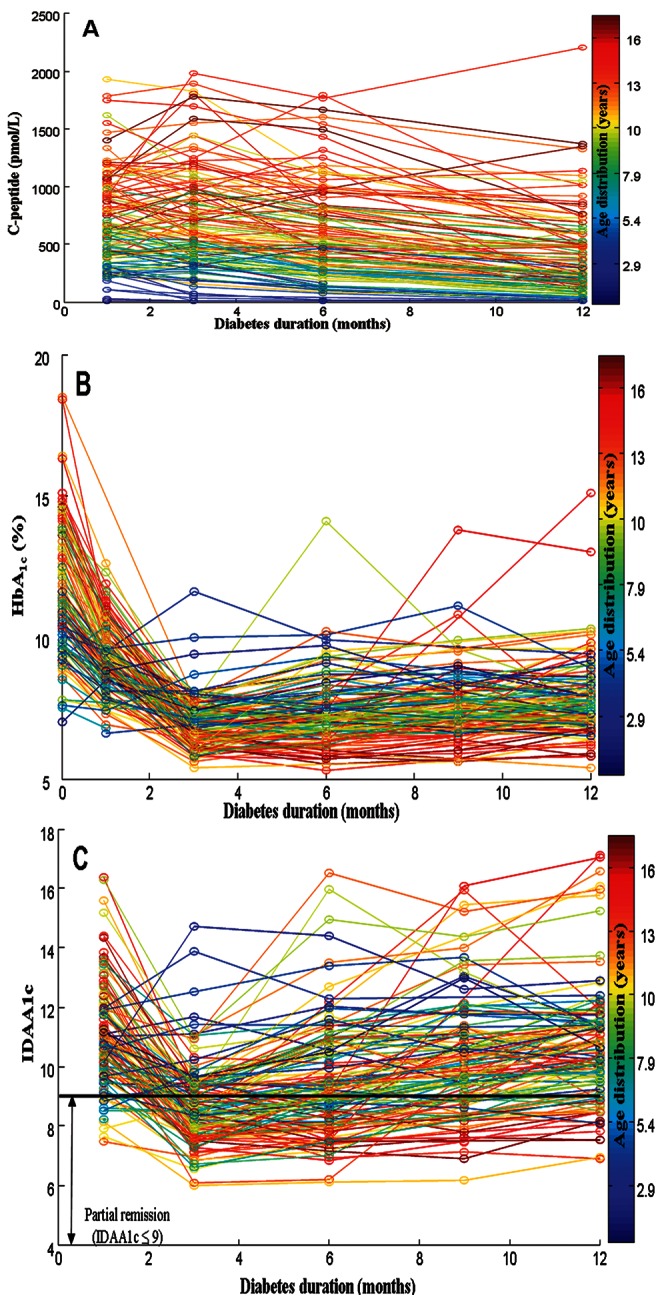
The course of stimulated C-peptide (pmol/L), HbA_1c_ (%) and IDAA1c for each child during the 12 months follow-up colored according to age. **A**. Raw values of stimulated C-peptide (pmol/L). Stimulated C-peptide was lowest in the youngest age groups. **B**. Raw values of HbA_1c_ (%). The HbA_1c_ level in the very young age group was lower at onset compared with the older age groups. **C**. Raw values of IDAA1c. The children with points below the black are in partial remission at that time point defined as IDAA1c ≤9. Very few of the very young children were in partial remission during the 12 months follow up (21.1% after 3 months).

The median level of **stimulated C-peptide** was unchanged from 1 to 3 months, but subsequently decreased from 3 to 12 months after clinical disease onset (Table 2). The association between age and stimulated C-peptide is shown in Figure 2A for each individual. Regression analysis revealed an increasing positive significant effect of age to stimulated C-peptide throughout the study period (P≤0.0001). Mean **HbA_1c_** and **IDAA1c** declined from disease onset, reached a nadir 3 months after diagnosis, and increased thereafter continuously throughout the study period (Table 2). The association between age and HbA_1c_ and IDAA1c, respectively, is shown in Figure 2B and 2C for each individual. Only 21.1% of the very young children (<5 years) were in partial remission (IDAA1c ≤9) 3 months after diagnosis, whereas 74.3% and 68.9% of the children in the two oldest age groups (5–10 years and >10 years) were in partial remission at this time point (P = 0.0002) (Figure 2C).

**Table 2:** Stimulated C-peptide (pmol/L), HbA_1c_ (%) and IDAA1c during 12 months follow up.

	1 month	3 mths	6 mths	9 mths	12 mths
Median C-peptide(range) (pmol/L)	628 (10–1934)	594 (10–1982)	490 (10–1797)	–	285.5 (10–2205)
Mean HbA_1c_ (SD) (%)	9.3 (±1.2)	7.0 (±0.86)	7.3 (±1.2)	7.5 (±1.2)	7.7 (±1.32)
Mean IDAA1c (SD)	11.0 (±1.7)	8.7 (±1.4)	9.5 (±1.9)	10.3 (±1.9)	10.7 (±2.0)

### Latent Factor Modelling (multi-block analyses)

The three data blocks were modelled by a two component multi-block model describing 21.6% of the total variation in the block of dynamic paraclinical biomarkers. These components were assigned ‘β-cell function’ and ‘ZnT8Ab’. The first component reflects data from all three data blocks, whereas the second component only reflects data from the dynamic biomarker- and gene blocks.

### Component 1: Identification of Baseline Clinical Characteristics and Genetic Profiles Related to Declining ‘β-cell Function’ (dynamic paraclinical biomarker block)

**Figure 3:**
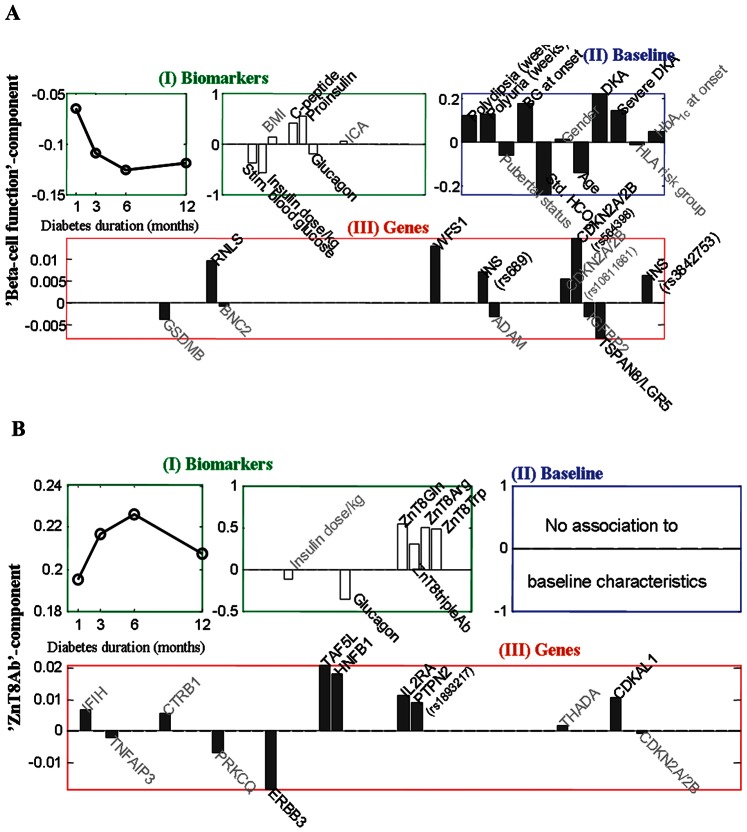
**Multi-block analyses: A. ‘β-cell function’-component: (I)** Pattern of the paraclinical biomarkers forming the ‘β-cell function’-component and the progression of this biomarker pattern during the first 12 months after diagnosis. (**II**) The pattern of baseline (the time of diagnosis) characteristics predictive for the biomarker pattern of the ‘β-cell function’-component over time (p = 0.001), were long duration of symptoms, younger age and DKA and consequently high blood glucose and low level of standard bicarbonate. (**III**) The pattern of type 1- and T2D associated SNPs associated with the biomarker pattern of the ‘β-cell function’-component over time (I) (p = 0.006) and the pattern of baseline characteristics in (II). The best genetic predictors for the biomarker pattern of the ‘β-cell function’-component over time were a combination of more risk alleles of the *INS (*rs689 and rs3842753), *RNLS* (rs10509540), *WFS1* (rs10010131) and *CDKN2A/2B* (rs564398) variants; and less risk alleles of the *TSPAN8-LGR5* (rs7961581) variant. **B. ‘ZnT8’-component:** (**I**) Pattern of biomarkers forming the ‘ZnT8-component’ and the progression of this biomarker pattern during the first 12 months after diagnosis. (**II**) This component was not significantly associated with baseline characteristics. (**III**) The pattern of T1D and T2D associated SNPs associated with the biomarker pattern of the ‘ZnT8’-component over time (I) (p = 0.0005). The best genetic predictors for the biomarker pattern of the ‘ZnT8Ab’-component were a combination of more risk alleles of the *IFIH1* (rs1990760), *TAF5L* (rs3753886), *HNF1B* (TCF2, rs4430796), *IL2RA* (rs11594656), *PTPN2* (rs1893217) and *CDKAL1* (rs10946398) variants; and less risk alleles of the *ERBB3* (rs2292239) variant.

The first association pattern from the multi-block analyses is shown in Figure 3A. This component describes a dynamic pattern of biomarkers related to declining β-cell function reflected as a decline of stimulated C-peptide and proinsulin in combination with an increase of stimulated blood glucose, glucagon and insulin dose per kg bodyweight during the first 12 months after onset (Figure 3A (I)). This component is denoted ‘β-cell function’. The ‘β-cell function’-component is associated with a pattern of baseline clinical characteristics consisting of young age, DKA, low standard bicarbonate, high level of blood glucose and duration of symptoms (polyuria and polydipsia) (Figure 3A (II)). The described association between the pattern of dynamic biomarkers and the corresponding pattern of baseline characteristics of the ‘β-cell function’-component are significant (P = 0.0004). Furthermore, the ‘β-cell function’-component is associated with a genetic pattern of multiple number of risk alleles of T1D associated SNPs from the insulin VNTR region (*INS* (rs3842753 and rs689) and the *RNLS* (rs10509540) together with T2D associated SNPs in *WFS1* (rs10010131) and *CDKN2A/2B* (rs564398) genes and protective alleles of the T2D associated SNP (rs7961581) in the *TSPAN8-LGR5* gene (P = 0.006) (Figure 3A (III).

### Component 2: Identification of Genetic Profiles Related to the Dynamic ZnT8Ab Profile

The second component, denoted ‘ZnT8Ab’-component, describes the development of ZnT8Abs (reflected as high levels of ZnT8Arg, ZnT8Trp, ZnT8Gln and ZnT8TripleAb) combined with low levels of stimulated glucagon (Figure 3B). This pattern increases until 6 months after diagnosis, and subsequently stabilizes (Figure 3B (I)). The ‘ZnT8Ab’-component is strongly related to a genetic profile of risk alleles of SNPs in the T1D related genes: *TAF5L* (rs3753886), *HNF1B* (rs4430796), *IL2RA* (rs11594656) and *PTPN2* (rs1893217) in combination with the T2D associated SNP: *CDKAL1* (rs10946398); and protective alleles of the T1D associated gene *ERBB3* (rs2292239) (P = 0.0005) (Figure 3B (III)). The ‘ZnT8Ab’-component was not found to be correlated to age or any other of the baseline characteristics (P>0.5).

### External Validation of the Multi-block Model

In the split half analysis the components were compared between the two strata. Especially the biomarker profiles in relation to the ‘β-cell function’-component and ‘ZnT8Ab’-component, were consistent (P = 0.002 and P = 0.0001). The selection of genes in the split-half procedure were to some extend inconsistent (P = 0.2) but these analyses were especially hampered by the loss of modelling power by reducing the data with 50%. Comparison with a model based on the Hvidoere Cohort (n = 275) revealed, that the first component (‘β-cell function’) is consistent (P = 0.009), with a similar baseline pattern (P = 0.0028), including a fair overlap in genes selected for this component (P = 0.08). However, the pattern of the second component (‘ZnT8Ab’) was not strong enough to be revealed by a two component model based on the Hvidoere Cohort.

## Discussion

### ‘β-cell Function’-component

The present study applies *multi-block methodology* based on *Latent Factor Modelling* (appendix S1) with the aim of identifying associating clinical, paraclinical and genetic patterns in a well characterized cohort of children with newly diagnosed T1D. We suggest applying this modelling method to address one of the largest challenges in post-GWA studies, the association between disease-related risk loci and metabolic/clinical phenotypes. The first component extracted from the model, ‘β-cell function’, reflects a well-known pattern between stimulated C-peptide, age [1]–[3] and metabolic derangements [4]. When applying *multi-block analysis* we did, however, identify less well described relations between clinical observations, as duration of symptoms and decline of β-cell function, indicating the importance of early diagnosis of the disease. The cohesion between stimulated C-peptide and proinsulin is supported by previous reports [2], [18], [29]. We refine the dynamic pattern of declining β-cell function represented by stimulated C-peptide and proinsulin with the combination of increased stimulated blood glucose levels and daily insulin dose per kg [18].

This ‘β-cell function’-pattern is associated with a genetic profile of T2D related genes including multiple risk alleles of the SNPs in the *WFS1* and *CDKN2A/2B* [25] genes and protective alleles of the SNP in the *TSPAN8/LGR* gene [25], together with multiple numbers of risk alleles of the SNP in the T1D related gene *RNLS* [24] and the two SNPs (rs3842753 and rs689) from the insulin *VNTR* region. The SNPs from the insulin *VNTR* region has been documented to contribute to T1D susceptibility [30] and preservation of β-cell function [9] supporting the present findings, whereas the relation of the other genes with the residual β-cell function is novel. The HLA risk genotypes were not present in the genetic pattern of the ‘β-cell function’-component, although they are important determinants of earlier disease onset [31]. HLA genotypes do, however, not seem to predict residual β-cell function [2] and, thus, we would not expect a contribution of the HLA genotypes in the genetic pattern of the ‘β-cell function’-component model. The ‘β-cell function’-component is estimated between all three data blocks (biomarkers, baseline characteristic and genetic background), whereby the genetic fingerprint also associates to the described pattern of baseline characteristics. From this analysis it is not possible to conclude whether the genetic background results in earlier disease onset, and only indirectly predicts a more rapid β-cell destruction, or if the genes alone reflect a certain profile of β-cell destruction. However, conventional regression analyses (data not shown) indicate that the genetic effect is mediated through younger age at onset, since the predictive C-peptide effect disappears when adjusting for age. Thus, multiple risk alleles of SNPs from the *WFS1, CDKN2A/2B* and *RNLS* genes may predict earlier onset of T1D probably due to a more rapid β-cell destruction following the first β-cell damage. These findings confirm a recent work by Howson et al [32], where polymophisms in the *RNLS* gene were associated with earlier onset of T1D.

### ‘ZnT8Ab’-component

The second component ‘ZnT8Ab’ describes a pattern of ZnT8Abs, which is negatively associated with glucagon levels. The positive association between the different ZnT8Abs is described earlier [6], [8], whereas the association between ZnT8Abs and glucagon is novel. A significantly positive association between ZnT8Arg and stimulated C-peptide 3, 6 and 12 months after diagnosis has previously been found in the present cohort [8], being in accordance with other studies [6], [7]. The association pattern between stimulated C-peptide and the baseline characteristics is, however, much stronger, which is why C-peptide is not present in the ‘ZnT8Ab’-component. The association between ZnT8Abs and glucagon indirectly supports the association between ZnT8Arg and stimulated C-peptide and the intra-islet hypothesis [33], since high level of stimulated C-peptide combined with low level of stimulated glucagon may reflect an ongoing suppressive effect of local insulin directly on the α-cell [34]. Indeed the β-cell function declines approximately 50% from 1 to 12 months after diagnosis, while the α-cell function increase by 17% during the same period of time [34]. Furthermore, β-cell derived zinc has been shown to exert an inhibitory effect on glucagon secretion although controversy still exists [35]. As free zinc level is higher in ZnT8 over-expressing cells [36], high levels of ZnT8Abs may reflect a higher level of zinc in the islets. Thus, the findings in the present study support the hypothesis that glucagon secretion is sensitive to suppression by β-cell derived zinc in a complex interplay with other factors, although the exact role of ZnT8 in α-cells and also the ZnT8Abs remains uncertain.

The ‘ZnT8Ab’-component was associated with a genetic fingerprint based on SNPs closest to *TAF5L*, *HNF1B*, *CDKAL1* and *ERBB3* genes, which are all expressed in β-cells (www.t1dbase.org). No studies have found this combined genetic association with ZnT8Abs [37], but there are studies relating *TAF5L* [38] and *ERBB3* [39] to T1D and *CDKAL1* [40] and *HNF1B* [41] to T2D. We did not find the *SLC30A8* gene to be present in the genetic pattern of the ‘ZnT8Ab’-component, although we know there is a very strong correlation between the rs1326634 SNP of the *SLC30A8* gene and the subtype of ZnT8Ab, such that the CC genotype carriers have higher ZnT8Arg and vice versa [6], [8]. Since the ‘ZnT8Ab’-component describes a pattern of increasing levels of *both* the ZnT8Arg and ZnT8Trp autoantibodies, we would not expect to find an effect of the *SLC30A8* gene.

### Validation

As the statistically methods in the present study are novel the work is validated both by internal (permutation testing), simulated external (split half analysis), and true external (reproducibility in Hvidoere) validation. Internal validation is by far the most used validation method where a central statistics is tested based on the data from which it is estimated (e.g. the p-values for a correlation coefficient). A slightly more rigorous form is the use of a *simulated* external validation, where central statistic is estimated in a part of data, say 80%, and then tested in the remaining part (20%) and the most rigorous procedure is to try to reproduce the results in new independent data. The internal approach reveals pattern to pattern significant association for all components. The split half analysis further verifies that the biomarker profiles in relation to the ‘β-cell function’-component and ‘ZnT8Ab’-component, were consistent, whereas the selection of genes in the split-half procedure were to some extend inconsistent. However, the split-half procedure was especially hampered by the loss of modelling power by reducing the data by 50%. The external validation by comparison with a model based on an independent dataset (the Hvidoere Cohort) revealed, that the first component (‘β-cell function’) is consistent, whereas the pattern of the second component (‘ZnT8Ab’) was not strong enough to be revealed by a two component model based on the Hvidoere Cohort. However, only the ZnT8Trp and the ZnT8Arg are available in the Hvidoere Cohort, for which reason we cannot expect this component to be revealed in this cohort.

In conclusion, complex *Latent Factor Modelling* based on data from a clinically well characterized Danish cohort of children and adolescents with newly diagnosed T1D can describe disease progression patterns and identify biomarker-genetic interacting partners in complex data. Thus, the present study proposes a novel method for linking patterns of genetic risk loci with clinical phenotypes. The study also supports novel hypothesis linking some of the low-risk genes to earlier onset of T1D and indirectly to more aggressive β-cell destruction after the first autoimmune insult. Furthermore, these findings indicate association patterns for disease progression and generate new hypotheses, which need to be confirmed in other cohorts or tested in *in vitro* assays using cell culture systems. The unravelling and understanding of the natural history of T1D during the remission phase is crucial as a significant number of patients still retain a considerable residual β-cell mass at this time point and might therefore benefit from immunomodulatory intervention therapy enhancing β-cell survival and regeneration.

## Supporting Information

Appendix S1 **Further methodological details regarding the** ***Coupled Matrix Tensor Factorization*** **method.**
(DOC)
