# Supplementary material for: Complex Multi-Block Analysis Identifies New Immunologic and Genetic Disease Progression Patterns Associated with the Residual β-Cell Function 1 Year after Diagnosis of Type 1 Diabetes
Source: PLoS One. 2013 Jun 5;8(6):e64632. doi: 10.1371/journal.pone.0064632 (PMC3674006; doi:10.1371/journal.pone.0064632)
Supplement: Appendix S1 — Further methodological details regarding the Coupled Matrix Tensor Factorization method. (DOC) [file pone.0064632.s001.doc]

**Appendix S1**

*Coupled Tensor Matrix Factorization*

The three datasets analyzed in the present study are biomarkers evaluated over time (
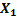
), cross- sectional baseline characteristics (
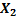
) and genetic polymorphisms (
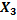
).


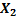
 and
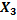
 can be arranged in a matrix with *n* rows (number of patients) and *p* columns (number of baseline characteristics or genetic polymorphisms).
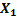
 has a higher order structure and can be arranged as a data cube often referred to as a tensor.
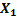
 has three dimensions, the number of samples (*n*), the number of time points (*q*) and the number of biomarkers (*p*). The three data blocks are jointly factorized such that


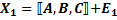


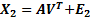


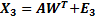


The factorization of
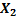
 and
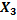
 has similar forms as common techniques such as Principal Component Analysis (PCA), Independent Component Analysis (ICA) etc. The factorization of
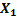
 is of similar form to PARAllel FACtor analysis (PARAFAC) / CANDECOMP (50).

The factor scores (
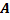
) describing the variations in distribution of the samples, are in this block formulation the *same* for the three blocks. This restriction makes the model different from e.g. PCA or PARAFAC on the three single blocks independently. The solution (estimates of the factor matrices
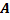
,
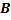
,
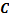
,
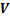
 and
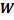
) are found by minimization of the sum of squared residuals across all blocks simultaneously and amounts to minimizing


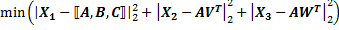
 [1]

In order not to favour single variables measured in a wide range, all variables are initially scaled to equal standard deviation. The individual blocks are likewise scaled to equal frobenious norm, such that each block of data is equally likely to be described by the model.

Constraints

In order to modify the block solution such that it is easier to interpret some constraints are put on the factor matrices. The unconstrained solution will to some extend describe all variables in all the components. However, some variables might only contribute slightly and could just as well be set to zero. We use an *L*_1_ norm constraint on the individual vectors of the factor matrix to impose sparse results (components with only a subset of the variables being described). We have chosen to make the factor matrixes related to the biomarkers (
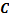
) and the genetic profiles (
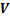
) sparse. The parameter was selected such that app. 50% of the biomarkers, and 30% of the genes are active in each component. The factor matrix describing the timely development of the biomarker patterns (
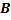
) was constrained to obtained all non-negative values. The optimization problem hence becomes


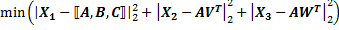
 [2]

Subject to:


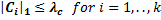


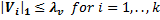


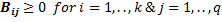


Where
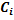
 and
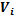
 refers to the columns of
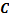
and
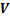
respectively and
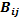
 correspond to the individual elements of
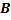
. *k* is the number of components, and *q* the number of time points.

Internal validation by permutation testing

Factor models can be estimated on totally random data and lead to spurious results. It is hence of utmost importance to validate the model in order to ensure that interpretation is done on robust results. The multi-block model aims at extracting common patterns from different sources of information such that a pattern of baseline characteristics is associated with a pattern of biomarkers over time. In order to estimate the significance of such an association a random permutation test procedure is applied where, in one block of data, the patients are randomly permuted.

We use a permutation testing approach to evaluate the individual block pattern associations.

Consider for example component one, which reflects a pattern both in the biomarker, gene and baseline block. The permutation procedure aims at corrupting the data block association via randomly permuting the rows of one of the matrices, then a model is estimated, and the association between the block components is estimated (as a correlation). This *null* association is then compared with the association observed for the true model. If the true association is close to the *null* association, then this is assumed to be spurious. In total 10.000 random permutations are performed.

Computation and numerical issues

The algorithm for solving the constrained CMTF problem is described in Rasmussen *et al* (un published). The code is written in Matlab® R2011a ver. 7.12.0.635. The object function (equation 2 above) is not strictly convex so there is no guarantee that an apparent solution is truly the global minimum. Be aware that this is the case for a number of models used within computational biology and statistics such as for example k-means clustering, PARAFAC, etc. and there is an entire scientific field dealing with global optimization of non-convex problems. In order to assure that a solution is indeed the global minimum several starts are conducted followed by evaluation of the corresponding solutions. That is: The initial parameter estimates are drawn from a random distribution and the subsequent solution is evaluated in terms of loss (sum of squared errors). A solution is valid if it is reproducible (obtained from several different random initializations) and the minimum across a number of solutions.

It is registered that for the present data 99 out of 100 random initializations lead to the same global optimum solution.
